# Joint Action European Partnership for Action Against Cancer

**DOI:** 10.1186/0778-7367-70-24

**Published:** 2012-10-24

**Authors:** Marjetka Jelenc, Elke Van Hoof, Tit Albreht, Matic Meglič, Marija Seljak, Sandra Radoš Krnel

**Affiliations:** 1 National Institute of Public Health, Trubarjeva 2, 1000, Ljubljana, Slovenia; 2 Belgian Cancer Center, Scientific Institute of Public Health, Brussel, Belgium; 3 Experimental and Applied Psychology, Faculty of Psychological and Educational Sciences, Vrije Universiteit Brussel, Brussel, Belgium

**Keywords:** EPAAC, Cancer, Health policy, Public health, Joint Action

## Abstract

Cancer is a major European public health issue and represents the second most important cause of death and morbidity in Europe. Moreover, as a result of constant advances in medicine, medical technology and other sciences, and due to improvements in economic circumstances, cancer survival rates are increasing in Europe and prevalent cases (i.e. number of subjects who have experienced cancer) represent a growing proportion of the population. In order to tackle cancer efficiently throughout the European Member states, the European Commission launched the Joint Action (JA) ‘European Partnership for Action Against Cancer’ (EPAAC) facilitated by the Community Health Programme, in September 2009. EPAAC is designed to fill a gap in cooperation, collaboration and shared experiences for countries with similar needs and diverse experience in the area of their national cancer control policies. Activities and studies are tackling the main challenges of cancer control in Europe as a whole and in the Member states, including the provision of services and health system responses, human resources and research. In contrast with previous European actions in the field of cancer, EPAAC joins different partners and stakeholders at various levels ranging from Member states (including Iceland and Norway) and Regions to patient representatives.

## Background

Modern-day health systems are under enormous pressure in terms of disease burden, demographic trends, the evolving roles of citizens, patients and health professionals, political challenges and sustainable financing. The twentieth century saw a dramatic epidemiologic shift in Europe and in the Western world in general, where infant mortality and deaths from communicable diseases such as tuberculosis, polio and infectious outbreaks were gradually overcome or controlled by effective public health policies. The most destructive diseases in Europe today are chronic rather than acute, affecting an ageing and predominantly urban population. Furthermore, older population is not only more susceptible to disease (and therefore more dependent on the health system), but also less able to contribute to already cash-strapped governments which are the main source of health system financing. In addition to this, with the ageing of the population and therefore of cancer patients, comorbidities have become much more frequent and this limits the use of the existing cancer-related guidelines or, at least, reduces their effectiveness. The unification of Europe has brought with it both challenges such as the harmonization of European legislation and standards, as well as opportunities in fields such as medical and health-system research. Finally, the predominance of chronic disease has made the role that citizens and patients play in managing their own health an indispensable one, while the globalized communication revolution has equipped them with the tools necessary to make their voices heard clearer than ever.

Cancer survival is increasing in Europe as a result of early diagnosis and improved treatment [1], with the corollary that the proportion of people in the population with a (past) diagnosis of cancer is growing. According to the project Surveillance of Rare Cancers in Europe (RARECARE) estimates – based on cancer registry data – in the European Union there were 3,566 individuals per 100,000 with a previous diagnosis of some form of cancer on 1st January 2003 equivalent to a total prevalence of nearly 17.8 million [2]. The number of prevalent cancer cases is projected to increase as the European population continues to age, as cancer incidence increases, and as survival improves [3]. European Cancer Registry-based Study on Survival and Care of Cancer Patients (EUROCARE) estimated that the proportion of patients (diagnosed from 1988 to 1999) considered cured of their cancer (all cancers combined) varied between 38% and 59% in women, and 21% and 47% in men, depending on the country [4].

Cancer incidence is rising in ageing populations and patients are increasingly informed, empowered and assertive with regard to their rights and their wishes. At the same time cancer is an umbrella term for 150 different pathologies which tax financial and human resources across multiple health services, from primary prevention to palliative care and rehabilitation. European governments are addressing these challenges as they can, with greater or lesser success, but it is apparent that effective and cost-efficient system-wide policies for cancer control are needed more urgently now than ever throughout the European continent.

The cancer burden also varies quite significantly by country, although admittedly completely accurate data remains elusive given the differences in reporting quality. Incidence in Hungary and the Czech Republic is notably higher than in the rest of the European Union, including neighbouring countries in Eastern Europe. Some northern European countries, such as Denmark and The Netherlands, also stand out for their high reported incidence.

Perhaps more illustrative of the scope of the cancer burden, however, is the mortality rate in comparison to other causes of death. Overall, it is a leading cause of death in the European Union, second only to diseases of the cardiovascular system. Moreover, in certain developed countries including France, Spain and The Netherlands, cancer kills more people than any other cause [5].

For many decades a lot of resources have been invested into trying to tackle cancer and numerous projects have contributed to the fight against this disease. Many projects are still ongoing.

The European Partnership for Action Against Cancer (EPAAC), launched in 2009, is a three-year initiative under the umbrella of the European Commission filling a gap in cooperation, collaboration and shared experiences for countries with similar needs and diverse experience in the area of their national cancer-control policies. EPAAC tackles the main challenges of cancer control in Europe as a whole and in the Member states, including service provision and health-system responses, human resources and research [6,7].

### EU public health strategy

In accordance with the Second Health Programme (SHP) and Annual Work Plan (AWP), EPAAC JA is contributing to the protection of the health and safety of individuals through its actions with regard to cancer, one of the eight leading causes of mortality and morbidity from non-communicable diseases in the World Health Organization (WHO) European Region. Demographic ageing is a global trend; the risk of cancer significantly increases with increasing age [8]. By 2030 it is estimated that there will be 75 million people living with cancer diagnosed within the previous five years, and almost 20 million of them will be living in the 53 countries of the WHO European region. While increased longevity is a great achievement, it is also a formidable challenge for both public and private budgets, for public services and for older people and their families. The expected increase in the incidence of cancer and the associated functional frailty associated with greater age require new approaches [9].

During the United Nations Summit on Non-Communicable Diseases (NCDs) in September 2011, the specific target of a 25% reduction in deaths from non-communicable disease by 2025 was set. Cancer is the cause of a significant number of these deaths [10].

### European Partnership for Action Against Cancer (EPAAC)

EPAAC embodies cooperation and collaboration in the tackling of cancer. It includes 38 Associated Partners from Health Authorities, Research Institutes and Patients’ Associations and over 90 collaborating partners. The National Institute of Public Health in Slovenia has assumed the role of leader of the EPAAC Joint Action.

Technically, EPAAC is a JA, facilitated by the Community Health Programme and has been created to contribute to the long-term aim identified by policy makers of reducing cancer incidence in Europe by 15% by 2020. In order to do so, EPAAC builds upon existing knowledge and expertise from ongoing or already completed projects. Consequently, the function of EPAAC is to integrate all existing knowledge in order to identify the most suitable practices. The specific challenge is to translate this existing knowledge into an information-support policy throughout the Member states. The second objective is to satisfy the need for an integrated and structured approach to tackle cancer in each Member state. For this purpose national cancer control programmes (NCCPs) preparation is being encouraged and NCCPs should be implemented in every Member state by 2013. Integrated NCCPs are public health programmes designed to ensure the coordinated and centrally managed implementation of evidence-based strategies for prevention, early detection, diagnosis, treatment, palliative care, rehabilitation and research for innovative solutions, as well as the evaluation of outcomes [11]. They are designed to help reduce the incidence, mortality and morbidity from cancer, as well as to improve the quality of life of cancer patients in all Member states throughout Europe.

Finally, a comprehensive European Cancer Information System (ECIS) is being designed.

ECIS involves all the institutions, individuals, procedures, and resources dealing with information and data about cancer and aims to provide the necessary knowledge to optimize cancer control and cancer research activities in Europe. Its main characteristics are:

Centralized data collection and database management.

Network-based data analysis and data quality control.

Serving as a research infrastructure for the cancer community at large.

Sustainable funding.

To make available and disseminate cancer burden indicators, the necessary indicators will be identified using European Cancer Health Indicators Project (EUROCHIP) and European Community Health Indicators (ECHI) programs. Population survival and sample data on staging and treatment acquired from cancer registries will be available through the EUROCARE project programme. Cancer Prevalence in Europe (EUROPREVAL) study will be used to provide the prevalence data. European-funded projects, such as EUROCHIP and Optimisation of the Use of Registries for Scientific Excellence in Research in Europe (EUROCOURSE), have identified the most important information needed to support policy action on cancer control, but such information is, however, still far from being available at a Europe-wide level [6].

Cooperation, collaboration and shared experience are key elements for EPAAC. In contrast to earlier projects, EPAAC brings together technical experts, scientists, stakeholders, patients’ representatives and policy makers in order to integrate all levels of expertise. In the future EPAAC proposes to disseminate existing knowledge and expertise as widely as possible, in order to harmonize the way cancer is dealt with and to achieve equality of care and survival throughout Europe.

### Work packages in EPAAC

In addition to work package on NCCPs, EPAAC consists of other four core work packages: Health promotion and prevention, Screening and early diagnosis, Healthcare, Research and Information and data. Each work package has its own strategic goals (Table 1).

**Table 1:** The content work packages and associated strategic goals of the EPAAC JA

**Work package**	**Main objective**
Health Promotion and Prevention	To raise awareness about cancer prevention among targeted groups in Europe
Screening and Early Diagnosis	Improvement of the implementation of the Council Recommendation on Cancer Screening of 2 December 2003
Healthcare	To identify, assess and exchange best-practices in cancer care across EU, including the patient’s perspective
Research	Development of a concerted approach for coordination of one third of research from all funding sources by 2013 in selected areas
Information and Data	To provide an analysis of the current status of cancer information in Europe and to propose, accordingly, a strategic reorganization of the data and information flow, aimed at improving cancer control support and cancer research activities.
National Cancer Plans	Preparation of guidelines for a high level standard NCP which includes the most significant areas

The work package on health promotion and prevention emphasizes improvement of the health status of young people and the promotion of a healthy lifestyle and a prevention culture – through the re-launching of the European Week Against Cancer (EWAC).

Work package on screening and early diagnosis will improve implementation of the Council Recommendation on Cancer Screening by alleviating key barriers, to make screening of appropriate quality, as recommended by the Council of the European Union, accessible to all citizens who may benefit. Further added value will be created by promoting synergy between cancer screening and other areas of early detection. A network of European Schools of Screening Management is initiated and dedicated to capacity building for implementation and improvement of population-based cancer screening programmes.

Work package on healthcare focuses on examples of good practices aimed at improving cancer care aligned with these elements across European health services, at national, regional and local level, which should be identified, and experiences exchanged. It has a particular focus on new organizational perspectives in cancer care, specifically networks of cancer care at regional level and for low frequency tumours and on issues of implementation of evidence-based guidelines in cancer for managers and clinicians. The key focus will be team working across these disciplines. A training strategy to improve psychosocial and communication skills among health care providers is being implemented.

Work package on research focuses on the development of a concerted approach to achieve coordination of one third of research from all funding sources by 2013 within selected areas of cancer research.

Special attention is given to centralising all existing data and research concerning cancer. Efficient policy indicators are envisaged to improve support policies addressing active and healthy ageing, and dealing with the impact of cancer throughout Europe. EPAAC will bring together all the existing European partners working on cancer burden indicators to coordinate the analysis and provision of information concerning incidence, mortality, survival and prevalence. Updated incidence and mortality data will be made available in the framework of the current activities of the International Agency for Research on Cancer (IARC) and of the European Network of Cancer Registries (ENCR), important co-operators. IARC in consultation with ENCR and other structures will be involved in the identification of a panel of experts in population-based data analyses, projections and forecasting [6].

Every year an Open Forum is organized to discuss the activities within specific work packages. Open Forum is a high-level plenary of the European network of cancer experts, health professionals, policy makers, civil society, patient associations, academia and provides a platform for all the members of the partnership and other interested key stakeholders at the European level in order to deepen their understanding of the challenges of cancer across Member states. A dedicated website ( http://www.epaac.eu) centralizes all relevant information and achievements. Online social media are used to connect to different types of audiences.

### Where next?

By adopting the European 2020 goals, several voluntary targets for preventing and controlling NCDs (2010–2020) are being accepted. First, a reduction of 20% is envisaged by improving the approach to cancer. While this may seem overly optimistic goal, recent publications have re-dimensioned this objective looking at the reduction in cancer incidence by type. Cancer incidence varies greatly across Member states but especially across types of cancer [12]. Cancer includes many different pathologies with dramatic variations of incidence and prevalence in different populations, different causalities, treatment options and prognoses.

The most commonly diagnosed cancers in Europe are, in order of numerical importance, breast, prostate, colorectal and lung cancers and these accounted for about half of the 3.4 million new cases in 2008. The cancer mortality burden is dominated by the same cancer types [12], although in a different order: lung cancer is responsible for almost 20% of all cancer deaths, followed by colorectal (12%), breast (7.5%) and pancreatic (5.4%). The following cancers are together responsible for about 1.8%-5.1% of total cancer incidence: cancers of the pancreas (2.9% of all new cancers), uterus (cervix and body of uterus combined, 4.5%), stomach (4.9%), oral cavity and pharynx (1.8%), kidney (3.1%) and non-Hodgkin’s lymphoma (2.7%). A few relatively uncommon cancers are nevertheless quite significant in terms of mortality, namely pancreatic and stomach, responsible respectively for 5.4% and 7.2% of total cancer mortality.

The cancer burden also varies quite significantly by country, although admittedly completely accurate data remains elusive given the differences in reporting quality.

**Figure 1:**
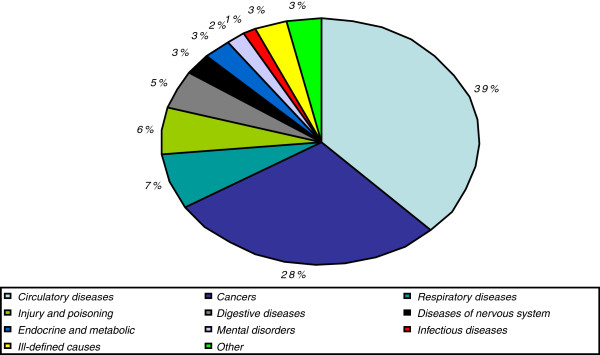
Proportional mortality by broad cause of death in the EU in year 2008.

Perhaps more illustrative of the scope of the cancer burden, however, is the mortality rate in comparison to other causes of death. Overall, it is a leading cause of death in the European Union, second only to diseases of the cardiovascular system (Figure 1).

In reaching the set goals, national plans are a key component for the planning and supervision of all actions undertaken. These national plans could also serve as support-policy development at national level in the Members states. A specific work package has gathered data on NCCPs throughout Europe. The analysis of these data has been finalised and will be presented to Member states by the end of 2012. Based upon these data and on already existing publications concerning NCCPs, guidelines and indicators are being identified. While there are different ways of understanding these programmes, and all are subject to structural and contextual peculiarities intrinsic to the diverse national settings, it is possible to sketch out the general characteristics of these policies.

All in all, the threat that cancer represents to the health of the population across the European Union is too serious to ignore. Primary and secondary prevention are essential in order to address incidence, but given that age is the greatest risk factor of all for developing these diseases, integrated care (including palliative and psychosocial aspects) must be improved. Research and professional training, the cornerstones of all medical progress, must drive improvements in service delivery across these areas. Health systems are faced with the same challenges in tackling cancer as when addressing a wide range of other health threats: achieving the overarching goals of improved general health, financial protection and response to citizen and patient needs.

Population-based information on cancer is fundamental for cancer-control activities, as well as for health-care research and, thanks to the existence of registries and to a long tradition of epidemiological research, is much more available than for most other diseases. In addition, very wide and high level research activity related to cancer information and data is ongoing in Europe. The considerable experience gathered and resources accumulated are not yet sufficiently deployed to achieve a proportional advance in knowledge in the field of cancer epidemiology. EPAAC work package 9 is developing proposals for the best use of these resources, in order to overcome the present fragmentation and duplication of effort. For this purpose the need has been recognized for the development of ECIS, intended to include all institutions, persons, procedures, and resources connected with information and data concerning cancer, and coordinated to provide the knowledge necessary to optimize cancer-control activities. ECIS should amalgamate and coordinate the entire process of data centralization, quality control, management, analysis and dissemination. It should allow public access to data, and also allow regulated but open access by the scientific community and other stakeholders to these data. In order to work as efficiently as possible, ECIS activities should combine the existing resources and experience of the European Institutions already involved in cancer information and data dissemination, most of which already have the knowledge, skills and instruments to carry out the main tasks required.

The Joint Research Centre (JRC), the European Commission in-house science service, would be the most appropriate to take on the task of hosting and managing the central data repository. ENCR could play a crucial role in maintaining a close connection between the ECIS activities and those of the participating cancer registries. IARC should continue to exercise the function of accreditation of cancer registries, training and provision of comparable cancer information at the global level. A network of European scientific institutions (such as those involved in EUROCARE or EUROCOURSE) is the most appropriate organizational structure to carry out the activities of data quality control, analysis, diffusion and dissemination in an efficient way [6].

## Competing interests

The authors declare they have no competing interests.

## Authors contributions

MJ drafted an initial version, EVH revised and edited the paper, TA, MM, MS and SRK reviewed the document. All authors read and approved the final manuscript.
